# SUPPORTING AN OLDER WORKFORCE BY CREATING AGE-FRIENDLY WORKPLACES: INTRODUCING A NEW MULTIDIMENSIONAL MODEL

**DOI:** 10.1093/geroni/igz038.693

**Published:** 2019-11-08

**Authors:** Raphael Eppler Hattab, Ilan Meshoulam, Israel Doron

**Affiliations:** University of Haifa, Israel, Israel

## Abstract

Creating age-friendly workplace environments is considered a central organizational approach for addressing the challenges of supporting an aging and older workforce. However, there are no concrete definitions or theoretical frameworks that explain the full meaning, assumptions and basic processes of this concept. This paper critically reviews the conceptualizations of the age-friendly workplace in the fields of organizational psychology and gerontology, and proposes (a) a new working definition of the concept, and (b) a multidimensional model that consists of a typology of age-friendly dimensions, representing the implications of human resource policies and practices that demonstrate the ways in which organizational climate and organizational culture support aging workers. This framework enables a better understanding of the organizational-occupational realities within an aging and older labor market, and thus serves as an effective foundation upon which future organizational measurements can be constructed. This presentation is based on the article: Eppler-Hattab, R., Meshoulam, I., & Doron, I. (2019). Conceptualizing Age-Friendliness in Workplaces: Proposing a New Multidimensional Model. The Gerontologist. 10.1093/geront/gny184.

